# Eugenol inhibits oxidative phosphorylation and fatty acid oxidation via downregulation of c-Myc/PGC-1β/ERRα signaling pathway in MCF10A-ras cells

**DOI:** 10.1038/s41598-017-13505-x

**Published:** 2017-10-10

**Authors:** Xianxin Yan, Guijuan Zhang, Fengjie Bie, Yanhong Lv, Yi Ma, Min Ma, Yurong Wang, Xiaoqian Hao, Naijun Yuan, Xuefeng Jiang

**Affiliations:** 1 College of Traditional Chinese Medicine, Jinan University, Guangzhou, China; 2 The School Outpatient Department, the First Affiliated Hospital of Jinan University, Guangzhou, China; 3 Bio-engineering institute of Jinan University, Guangzhou, China

## Abstract

Alteration in cellular energy metabolism plays a critical role in the development and progression of cancer. Targeting metabolic pathways for cancer treatment has been investigated as potential preventive or therapeutic methods. Eugenol (Eu), a major volatile constituent of clove essential oil mainly obtained from Syzygium, has been reported as a potential chemopreventive drug. However, the mechanism by which Eu regulates cellular energy metabolism is still not well defined. This study was designed to determine the effect of Eu on cellular energy metabolism during early cancer progression employing untransformed and H-ras oncogene transfected MCF10A human breast epithelial cells. Eu showed dose-dependent selective cytotoxicity toward MCF10A-ras cells but exhibited no apparent cytotoxicity in MCF10A cells. Treatment with Eu also significantly reduced intracellular ATP levels in MCF10A-ras cells but not in MCF10A cells. This effect was mediated mainly through inhibiting oxidative phosphorylation (OXPHOS) complexs and the expression of fatty acid oxidation (FAO) proteins including PPARα, MCAD and CPT1C by downregulating c-Myc/PGC-1β/ERRα pathway and decreasing oxidative stress in MCF10A-ras cells. These results indicate a novel mechanism involving the regulation of cellular energy metabolism by which Eu may prevent breast cancer progression.

## Introduction

Breast cancer is the most commonly diagnosed cancer and the second leading cause of cancer-related deaths in women^1^. According to the American Cancer Society, 231,840 new cases of invasive breast cancer were expected to occur among US women and 40,290 patients would die of breast cancer in 2015^1^. Approximately 30 in every 100,000 women will develop breast cancer in their lifetime in China and this proportion is increasing as the disease in younger patients becomes more common^2,3^. Theoretical advances over the past decades have indicated that the metabolic properties of cancer cells are greatly different from those of normal cells. In particular, altered cellular metabolism, a biochemical fingerprint of cancer cells, has been regarded as one of the”hallmarks of cancer”^4^. Research in cancer metabolism has traditionally focused on aerobic glycolysis, a phenomenon that rapidly proliferating tumor cells take up higher levels of glucose and that the majority of their glucose carbon is converted to lactate, even in the presence of oxygen (Warburg effect). The lowest yield of adenosine triphosphate (ATP) per glucose molecule is compensated by a higher glycolytic flux that results in a higher rate of ATP production during glycolysis compared to oxidative phosphorylation (OXPHOS)^5,6^. However, recent studies demonstrated that the percentage of glucose metabolized through glycolysis was decreased in the transformed MCF10 cells (MCF10A-ras) when compared to the nontransformed parental cells (MCF10-A). Conversely, flux through the tricarboxylic acid (TCA) cycle was higher in the transformed cell lines^7^. Studies have shown that enhanced mitochondrial oxidative phosphorylation in human breast tumors is a common feature, which allows epithelial cancer cells to produce high amounts of ATP in response to effectively promote tumor growth^8,9^. Furthermore, Lipid metabolism is also altered in rapidly proliferating cells. Breast cancer uses fatty acid oxidation (FAO) as an important energy source, which are proposed to provide ATP for survival and proliferation^10^.

This aberrant metabolic status of cancer cells has been seen as a side effect of alterations of signaling pathway due to proto-oncogenes for many years. However, a growing body of evidence suggests that activated oncogenes directly regulate cellular energy metabolism, hence causing tumorigenesis and allowing environmental change adaptation of transformed cells^11^. The c-Myc proto-oncogene may perform an important biological role in the tumorigenesis process, including proliferation, apoptosis, and differentiation^12,13^. One of the most important actions involves the metabolism process^14^. The c-Myc not only increases glycolysis in part through the regulation of lactate dehydrogenaseA (LDHA) and fatty acid oxidation (FAO), but also up regulates mitochondrial biogenesis to control cellular metabolism^15–17^. Peroxisome proliferator-activated receptor gamma coactivator-1-beta (PGC-1β) plays a critical role in regulating multiple aspects of energy metabolism^18,19^. It has recently been demonstrated that PGC-1β expression is up-regulated by c-Myc in breast cancer cells^20^. The estrogen-related receptor alpha (ERRα) functions downstream of the PGC-1β and controls the expression of genes involved in the TCA cycle, oxidative phosphorylation (OXPHOS), and lipid metabolism^20,21^. Therefore, the ability of c-Myc to regulate both glycolysis and mitochondrial activity is mediated by PGC-1β/ERRα signaling axis.

Eugenol (Eu,4-allyl-2-methoxyphenol), a phenolic natural compound which is the active component of Syzigium aromaticum (cloves), has been exploited for various medicinal applications such as antibacterial, antiviral, antioxidant, anti-inflamatory agent^22^. Furthermore, Eu has several anticancer properties in colon, liver, prostate and breast cancer^23–25^. Eu is suggested to prevent cancer progression through the modulation of expression of many proteins and genes involved in apoptosis, angiogenesis and cell growth^23,26^. However, the mechanisms in progression of breast cancer, and especially the effect of Eu on cellular energy metabolism in early breast cancer progression have not been understood. Therefore, it is important to determine if Eu alters cellular energy metabolism in cancer progression.

In the current study, MCF10A and H-ras transfected MCF10A (MCF10A-ras) breast epithelial cells were used as a model for carcinogenesis to examine the effect of Eu in regulation of cellular energy metabolism. The hypothesis of the current study is that Eu inhibits OXPHOS and FAO through downregulating c-Myc/PGC-1β/ERRα signaling pathway and decreasing oxidative stress in H-ras transfected breast epithelial cells but not in untransformed cells. These results will help to the understanding the role of Eu on regulation of cellular energy metabolism during mammary carcinogenesis.

## Results

### Eu suppresses cell growth and decreases intracellular ATP levels in MCF10A-ras cells but not non-cancerous MCF-10A cells

**Figure 1:**
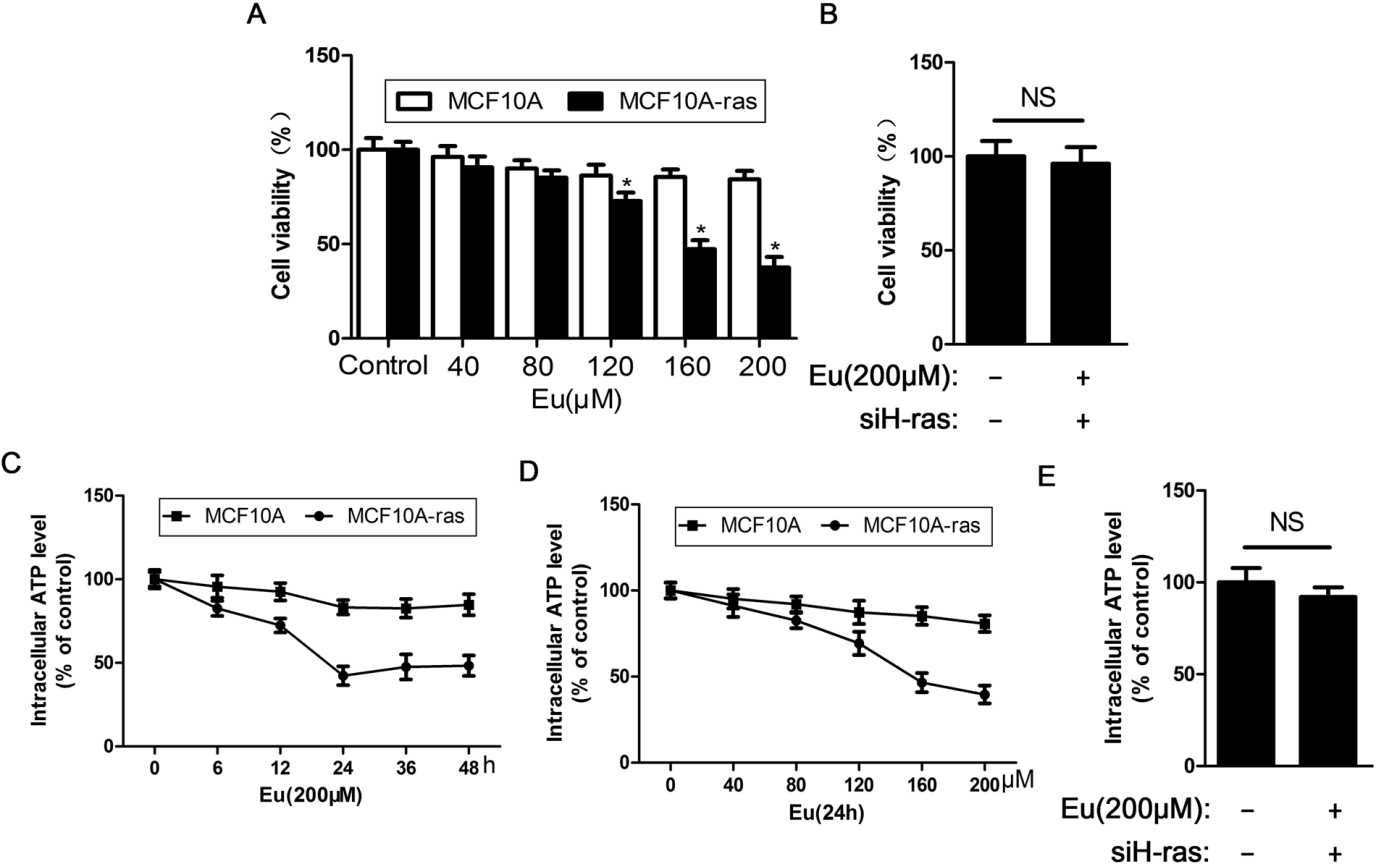
Eu inhibits cells proliferation and intracellular levels of ATP in MCF10A-ras cells but not non-cancerous MCF-10A cells. (**A**) MCF10A-ras or MCF-10A cells were treated with Eu for 24 h. (**B**) MCF10A-ras cells were transfected by siH-RAS, then the cells were treated with 200 μM Eu. Cell viability was determined by MTT assay. Error bars represent means ± S.D. *P < 0.05 versus non-treated control of MCF10A-ras cells. NS indicates no significant difference to control. (**C**,**D**) Cells were treated with 200 μM Eu for various time (0–48 h), or exposed to Eu at different concentrations for 24 h. (**E**) MCF10A-ras cells were transfected with siH-RAS, then the cells were treated with 200 μM Eu. Intracellular ATP levels were monitored using a luciferase-based assay. Error bars represent means ± S.D. NS indicates no significant difference to control. All experiments were repeated thrice.

The effects of Eu treatment on MCF10A-ras cells and non-cancerous MCF-10A cells was monitored by the MTT assay. As shown in Fig. 1A, Eu substantially inhibited MCF10A-ras cell growth in a dose-dependent manner, the viability of MCF10A-ras cells were reduced by 9.3%, 14.8%, 22.7%, 52.7% and 64.2% when MCF10A-ras cells were treated with 40, 80, 120, 160, 200 μM concentrations of Eu, respectively. However, Eu did not affect cell viability in MCF-10A cells which exhibited high resistance to Eu. This demonstrates that Eu has cytotoxicity against H-ras transfected breast epithelial cells, but its less toxic against non-tumorigenic breast epithelial cells. Next, to investigate the impact of Eu in the status of energy metabolism, we treated MCF10A-ras and MCF-10A cells with 200 μM Eu and tested intracellular ATP levels. As indicated in Fig. 1C, ATP levels were significantly decreased in MCF10A-ras treated with Eu, with a bottom level at 24 h, resulting in a 57.7% reduction. In contrast, Eu had a modest effect on MCF-10A, a control cell line. A dose response study (Fig. 1D) showed that decreased ATP levels were remarkably detected with as little as 40 μM Eu, reducing ATP levels to 91.2% relative to the control, with the lowest levels (39.6%) at 200 μM treatment in MCF10A-ras cells. Thus, 200 μM Eu will be adopted for subsequent research. Aberrant Ras activation and signaling can promote breast cancer development and growth^27^. To explore the role of H-ras in cell growth inhibition and decreased ATP levels induced by Eu, we use specific siRNA to down-regulate H-ras in MCF10A-ras cells. Interestingly, H-ras silencing remarkably restored Eu-induced inhibition of proliferation and ATP generation (Fig. 1B,E), indicating that Eu inhibited energy production via H-ras in MCF10A-ras cells.

### Eu inhibits c-Myc, PGC-1β and ERRα in cultured MCF10A-ras but not MCF-10A cells

**Figure 2:**
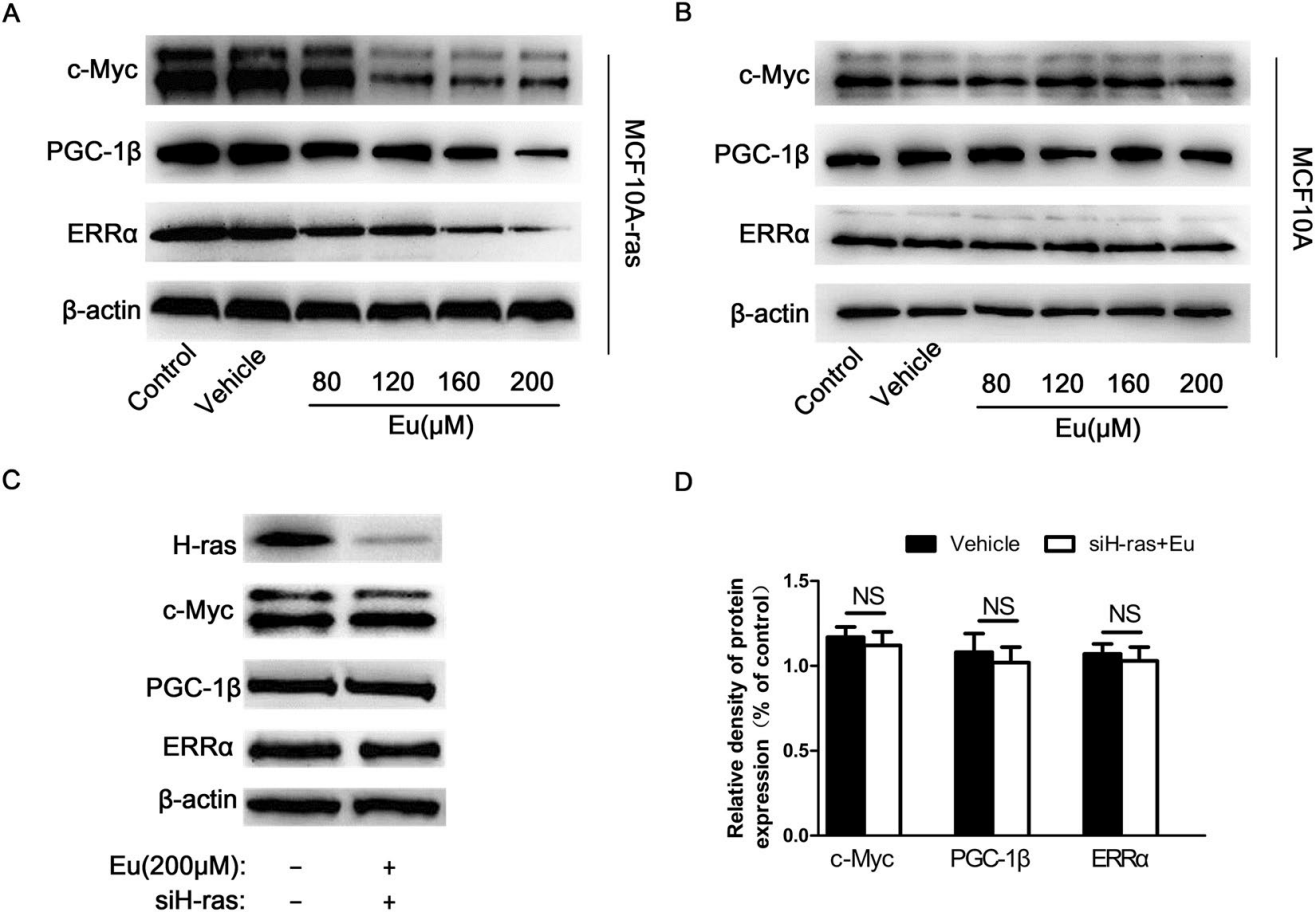
Eu suppresses the expression of several oncoproteins in cultured MCF10A-ras via H-ras but not MCF-10A cells. (**A**) MCF10A-ras or (**B**) MCF-10A cells were treated with either vehicle (DMSO) or various concentrations of Eu for 24 h. (**C**) MCF10A-ras cells were transfected with siH-RAS, then the cells were treated with 200 μM Eu. The protein expression of c-Myc, PGC-1β and ERRα were measured by western blot. β-actin was used as a loading control. (**D**) The Bars represent the quantification of the immunoblots for MCF10A-ras cells. Error bars represent means ± S.D. NS indicates no significant difference to control. Data are representative of three independent experiments. Full-length blots are presented in Supplementary Figs S1–S3.

The c-Myc, PGC-1β and ERRα are all crucial regulators of energy metabolism and play a key role in the pathogenesis of breast cancers^17,20^. To test whether Eu can inhibit c-Myc, PGC-1β and ERRα, MCF10A-ras and MCF-10A cells were treated with Eu for 24 h. As shown in Fig. 2A (also Supplementary Fig. S1), Eu significantly suppressed the protein expression of c-Myc, which was thought to contribute to breast cancer development and progression^28^, PGC-1β and ERRα in a dose-dependent manner, resulting in 18-63%, 21-68%, 24-71% reduction at various concentrations of Eu (80-200 μM) in MCF10A-ras cells, respectively. However, these proteins were not affected by Eu in MCF-10A cells (Fig. 2B, Supplementary Fig. S2). Ras is essential for Myc protein stability, and the co-expression of H-ras and c-Myc contributes to neoplastic transformation with early mammary carcinogenesis^29,30^. Therefore, we examined the status of c-Myc, PGC-1β and ERRα following the H-ras depletion in MCF-10A-ras cells treated with Eu. Notably, the deprivation of H-ras blocked the downregulated protein levels of the c-Myc, PGC-1β and ERRα induced by Eu (Fig. 2C-D, Supplementary Fig. S3), suggesting that Eu decreased the protein expression levels of the c-Myc, PGC-1β and ERRα via H-ras.

### Eu suppresses cell viability, ATP generation in MCF10A-ras cells by downregulating c-Myc and c-Myc-regulated expression of PGC-1β and ERRα

**Figure 3:**
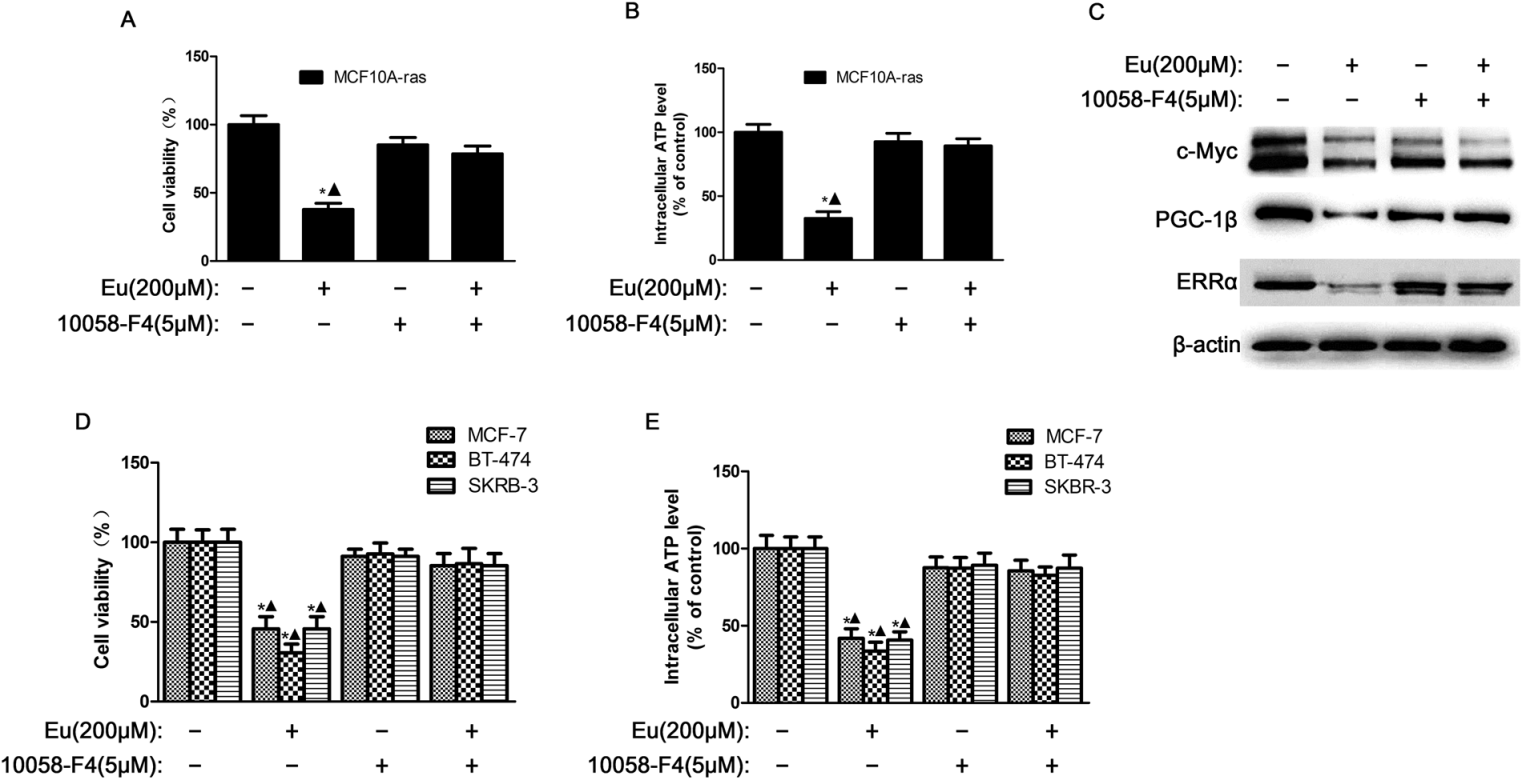
Eu suppresses cell viability, ATP generation in MCF10A-ras cells by downregulating c-Myc and c-Myc-regulated expression of PGC-1β and ERRα. MCF10A-ras, MCF-7, BT-474 and SKRB-3 cells were pre-treated with or without c-Myc inhibitor 10058-F4 (5 μM) for 1 h, followed by Eu (200 μM) treatment for 24 h. (**A**,**D**) Cell viability of MCF10A-ras, MCF-7, BT-474 and SKRB-3 cells with indicated treatments for 24 h was detected by MTT assay. (**B**,**E**) Intracellular ATP levels of MCF10A-ras, MCF-7, BT-474 and SKRB-3 cells were monitored using a luciferase-based assay, and (**C**) the protein expression of c-Myc, PGC-1β as well as ERRα were used for western blot analysis. ^*^ *P* < 0.05 compared with vehicle control group, ^▲^ *P* < 0.05 compared with 10058-F4 and Eu-treated control group. Data are representative of three independent experiments. Full-length blots are presented in Supplementary Fig. S4.

**Figure 4:**
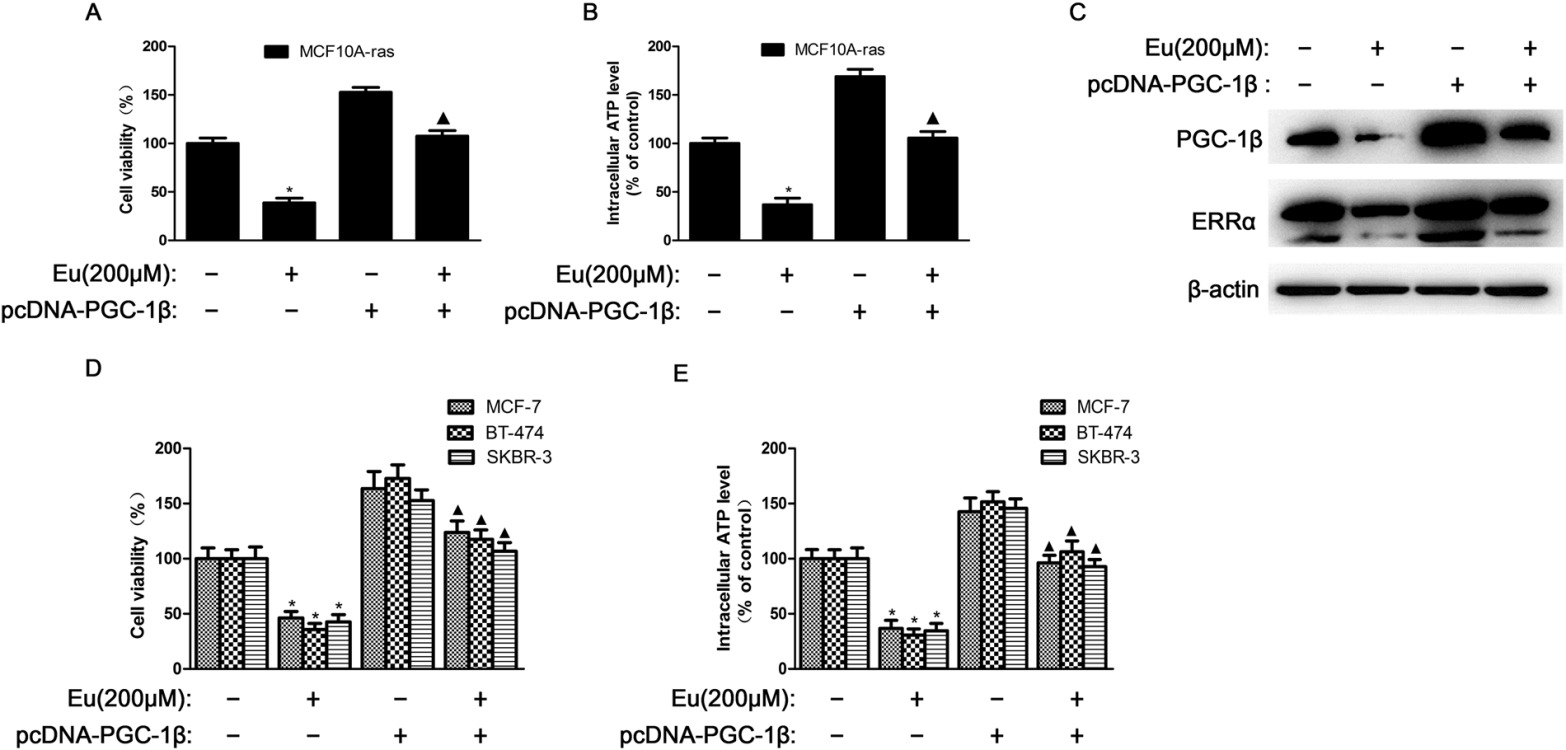
Eu suppresses cell viability, ATP generation in MCF10A-ras cells by downregulating PGC-1β and ERRα. PGC-1β overexpressed MCF10A-ras cells were then incubated with 200 μM Eu for 24 h. (**A**,**D**) Cell viability of MCF10A-ras, MCF-7, BT-474 and SKRB-3 cells with indicated treatments for 24 h was detected by MTT assay. (**B**,**E**) Intracellular ATP levels of MCF10A-ras, MCF-7, BT-474 and SKRB-3 cells were monitored using a luciferase-based assay, and (**C**) the protein expression of PGC-1β as well as ERRα were used for western blot analysis. ^*^ *P* < 0.05 versus vehicle control group, ^▲^ *P* < 0.05 versus PGC-1β overexpressed control group. The data were shown as the mean ± S.D. from three independent experiments. Full-length blots are presented in Supplementary Fig. S5.

The c-Myc is a proto-oncogene, which serves as a conduit for signaling events initiated at the membrane, facilitating PGC-1β expression and the induction of ERRα transcriptional activity^20^. To further probe regulatory relationships of Eu on c-Myc signaling cascades involving in metabolic adaptation, we treated MCF10A-ras cells with Eu for 24 h in the presence or absence of inhibitor of c-Myc, 10058-F4, which blocks the c-Myc/Max heterodimerization. As shown in Fig. 3C (Supplementary Fig. S4), Eu treatment robustly decreased the protein expression levels of c-Myc, PGC-1β and ERRα. The c-Myc inhibitor 10058-F4 apparently restored the Eu-induced inhibition of cell viability, ATP generation in response to Eu, indicating that Eu reduced energy generation via c-Myc in MCF10A-ras cells (Fig. 3A,B). These regulatory relationships are also operational in the MCF-7, SKBR3 and BT-474 breast cancer cell lines (Fig. 3D,E). Moreover, Inhibition of c-Myc activity by 10058-F4 restored the downregulated protein levels of the PGC-1β and ERRα induced by Eu (Fig. 3C, Supplementary Fig. S4). PGC-1β, a upstream target of ERRα, is key regulator of mitochondrial biogenesis and function, and fatty acid β-oxidation. we speculated that Eu may regulate energy metabolism through a process involving in PGC-1β. To test this idea, we constructed a pcDNA-PGC-1β overexpression vector. Western blot analysis indicated that PGC-1β overexpression led to increase in protein levels of ERRα (Fig. 4C, Supplementary Fig. S5). As shown in Fig. 4A–C, the inhibition of intracellular ATP production, cell viability and the regulation of ERRα caused by Eu were abolished by overexpression of PGC-1β. These effects can also be observed in MCF-7, SKBR3 and BT-474 breast cancer cells (Fig. 4D,E). The results confirm that Eu inhibits cell viability and ATP production by repressing the expression of c-Myc/PGC-1β.

### Eu inhibits OXPHOS in MCF10A-ras cells by downregulating PGC-1β/ERRα axis

**Figure 5:**
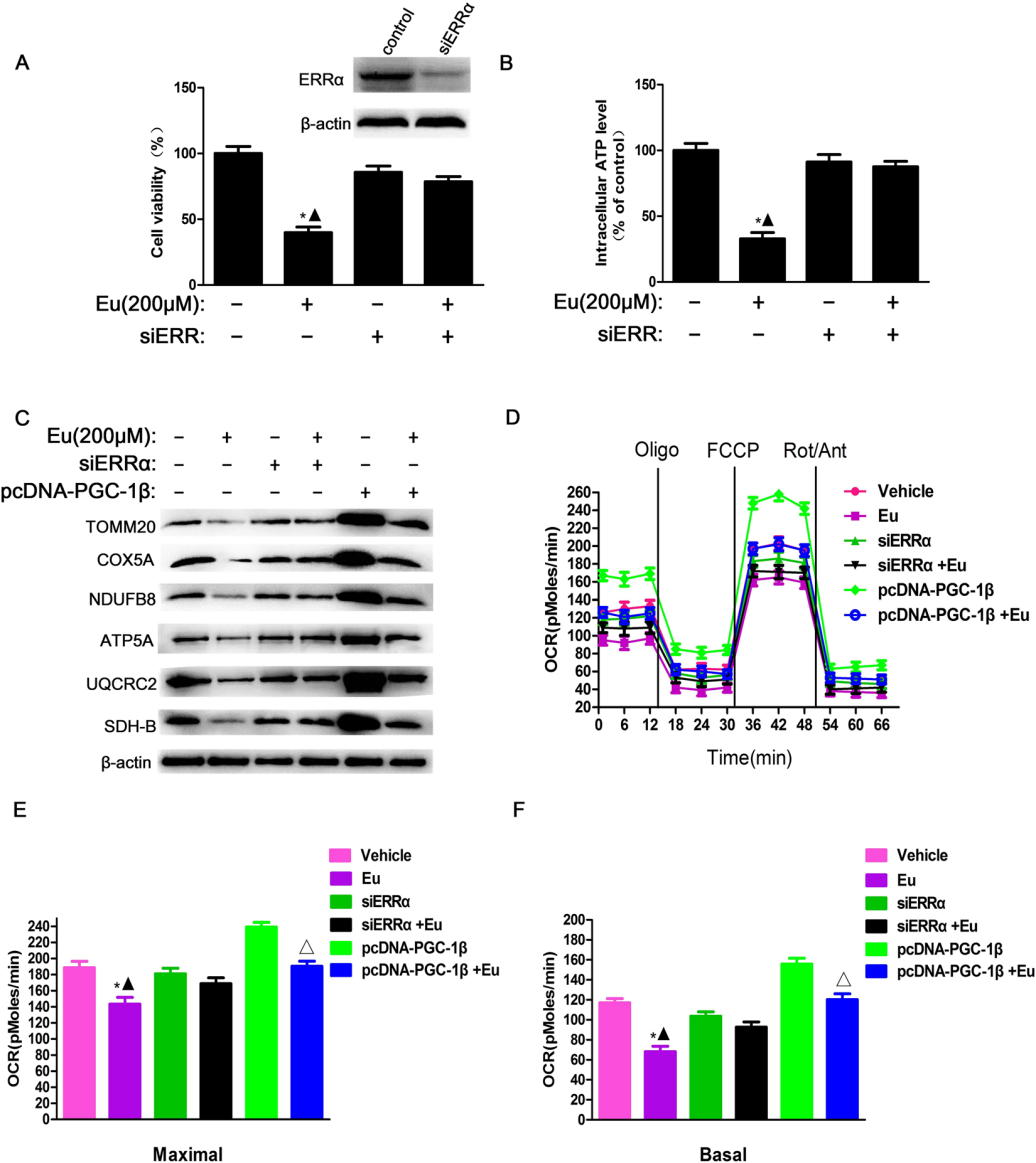
Eu inhibits OXPHOS in MCF10A-ras cells by downregulating PGC-1β/ERRα axis. MCF10A-ras cells were transfected with ESRRA siRNA, and then 200 μM Eu was added to MCF10A-ras cells for 24 h. In some experiments, MCF10A-ras cells were transfected with pcDNA-PGC-1β, followed by Eu treatment for 24 h. Cell viability (**A**) and intracellular ATP levels (**B**) were assayed as above. (**C**) The protein expression of mitochondrial complexes (COX5A, NDUFB8, ATP5A, UQCRC2, SDH-B) and TOMM20 with indicated treatments (Vehicle, Eu, siERRα, siERRα + Eu, pcDNA-PGC-1β, pcDNA-PGC-1β + Eu) was detected by western blot. (**D**) Cellular respiration of MCF10A-ras cells treated with indicated treatments was analyzed by mitochondrial stress test using a Seahorse XF96 Analyzer. (**E**,**F**) Basal and maximal respiratory rate were calculated from the OCR traces corresponding to representative graph as depicted in D. Data was shown as the mean ± S.D. for three independent experiments. *P < 0.05 compared with vehicle control group, ^▲^ *P* < 0.05 compared with siERRα and Eu-treated control group, ^△^ *P* < 0.05 compared with PGC-1β overexpressed control group. Full-length blots are presented in Supplementary Fig. S6.

ERRα plays a key role in energy homeostasis and will probably be targeted for the treatment of metabolic disorders, which controls the expression of genes involved in the glycolytic pathway, TCA cycle and OXPHOS^31,32^. To determine if ERRα is involved in metabolic inhibition elicited by Eu, we measured intracellular ATP levels and cell viability. Silencing of ERRα expression by a specific siRNA blocked Eu-induced inhibition of intracellular ATP levels and cell growth (Fig. 5A,B). Cellular proteins were extracted and subjected to western blot analysis for various OXPHOS components. As indicated in Fig. 5C (Supplementary Fig. S6), compared with the vehicle, the protein expression of mitochondrial complexes I (NDUFB8), II (SDH-B), III (UQCRC2), IV (COX5A) and V (ATP5A) and mitochondrial biogenesis marker (TOMM20) were respectively decreased by 78.2%, 72.6%, 76.0%, 81.5%, 53.7% and 67.3% in MCF10A-ras cells treated with 200 μM Eu. However, these effects were blocked by siRNA-mediated silencing of ERRα. As PGC-1β is a major regulator of ERRα activity in breast cancer cells, which stimulates mitochondrial biogenesis and respiratory function. To explore the role of PGC-1β in induction of OXPHOS, we stably overexpressed PGC-1β in MCF10A-ras cells, which were established molecules that drived mitochondrial biogenesis and increased OXPHOS. As expected, western blot assay showed that the mitochondrial complexes (I, II, III, IV and V) and TOMM20 were increased in PGC-1β-overexpressing MCF10A-ras cells, and these effects were nearly reversed by treatment with Eu (Fig. 5C, Supplementary Fig. S6). To further investigate the effect of Eu on mitochondrial respiration in MCF10A-ras cells, we measured the basal and maximal oxygen consumption rate (OCR) in response to modulators of electron transport chain (ETC) function using the Seahorse XF96 extracellular flux analyzer (Fig. 5D). As shown in Fig. 5E,F, Eu significantly decreased the basal and maximal OCR in MCF10A-ras cells. Knockdown ERRα with siRNA nearly restored any Eu-induced decrease in OCR and the inhibition of OCR induced by Eu were abolished by overexpression of PGC-1β. Thus, we conclude that Eu downregulates overall OXPHOS activity through inhibiting PGC-1β/ERRα axis in MCF10A-ras cells.

### Eu inhibits FAO in MCF10A-ras cells by downregulating PGC-1β/ERRα axis

**Figure 6:**
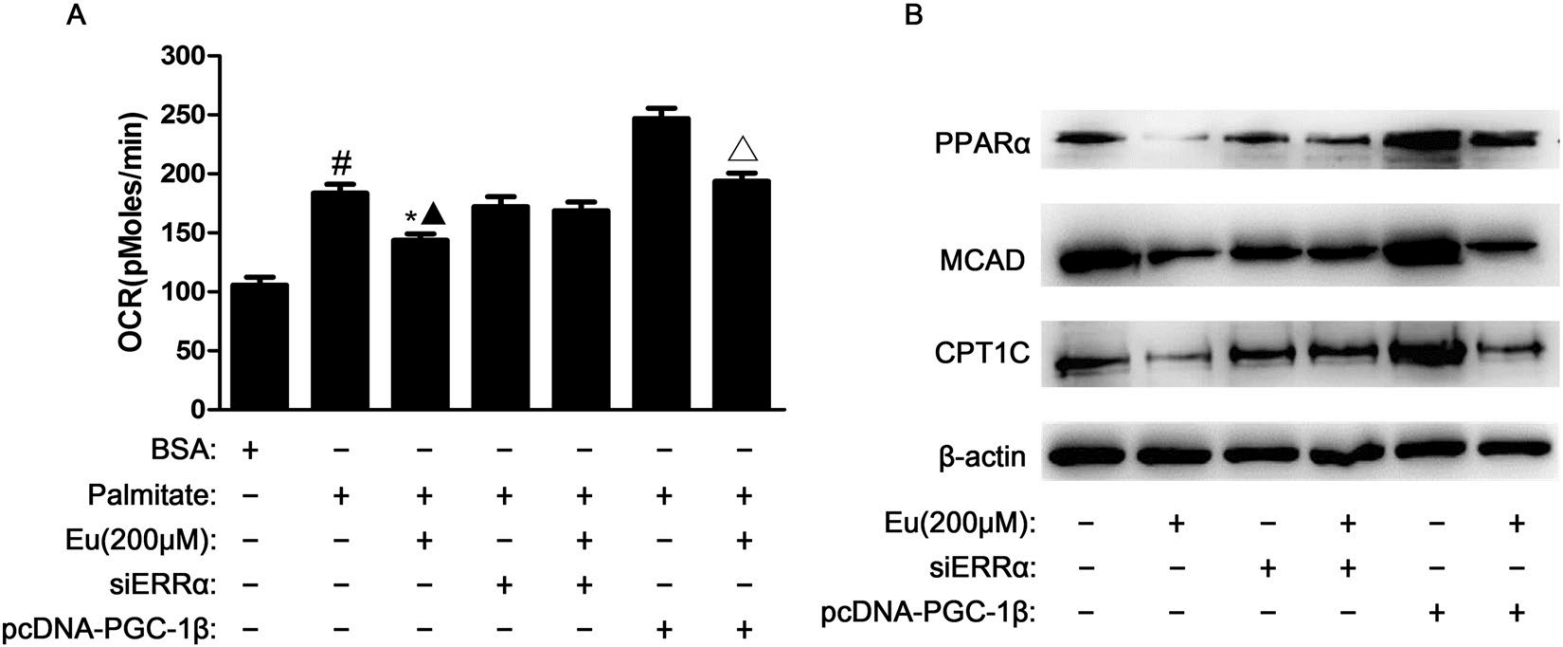
Eu inhibits FAO in MCF10A-ras cells by downregulating PGC-1β/ERRα axis. MCF10A-ras cells were transfected with ESRRA siRNA, and then 200 μM Eu was added to MCF10A-ras cells for 24 h. In some experiments, MCF10A-ras cells were transfected with pcDNA-PGC-1β, followed by Eu treatment for 24 h. (**A**) Fatty acid oxidation assay. Oxygen consumption rate was measured after 200 μM palmitate injection over 2 to 3 h and cells treated with BSA were served as a negative control. (**B**) Cellular proteins were extracted and subjected to western blot analysis for PPARα, MCAD, and CPT1C. ^#^ *P* < 0.05 compared with vehicle control group, *P < 0.05 compared with palmitate control group, ^▲^ *P* < 0.05 compared with siERRα and Eu-treated control group, ^△^ *P* < 0.05 compared with PGC-1β overexpressed control group. Full-length blots are presented in Supplementary Fig. S7.

Fatty acids is a major source of cellular energy for cancer cells. Previous studies suggest that FAO can promote cancer cell survival in conditions of metabolic stress^33^. To this end, we measured the oxygen consumption rate (OCR). Addition of palmitate increases OCR because of increased substrate availability for FAO. We found that Eu significantly diminished OCR in the presence of palmitate in MCF10A-ras cells. PGC-1β overexpression nearly reversed FAO inhibition induced by Eu, indicating that Eu inhibited FAO via PGC-1β in MCF10A-ras cells (Fig. 6A). Moreover, Eu downregulated the expression of FAO proteins such as PPARα (peroxisome proliferator-activated receptor alpha), CPT1C (carnitine palmitoyltransferase 1 C), and MCAD (medium-chain acyl-coenzyme A dehydrogenase) which were directly regulated by ERRα, and the expression of these proteins were decreased by 89.6%, 63.7% and 79.3%, respectively. Overexpressing of PGC-1β restored the downregulated protein levels of FAO induced by Eu (Fig. 6B, Supplementary Fig. S7). To further verify these proteins are metabolic target proteins of ERRα in MCF10A-ras cells, we use specific siRNA to down-regulate of ERRα. As demonstrated in Fig. 6A,B, the inhibition of FAO and PPARα, MCAD, and CPT1C induced by Eu were reversed by siRNA-mediated silencing of ERRα. Taken together, these data strongly suggest that Eu regulates many proteins of fatty acid oxidative metabolism in MCF10A-ras cells by suppressing PGC-1β/ERRα axis.

### Eu inhibits OXPHOS and FAO in MCF10A-ras cells through decreasing 0xidative stress

**Figure 7:**
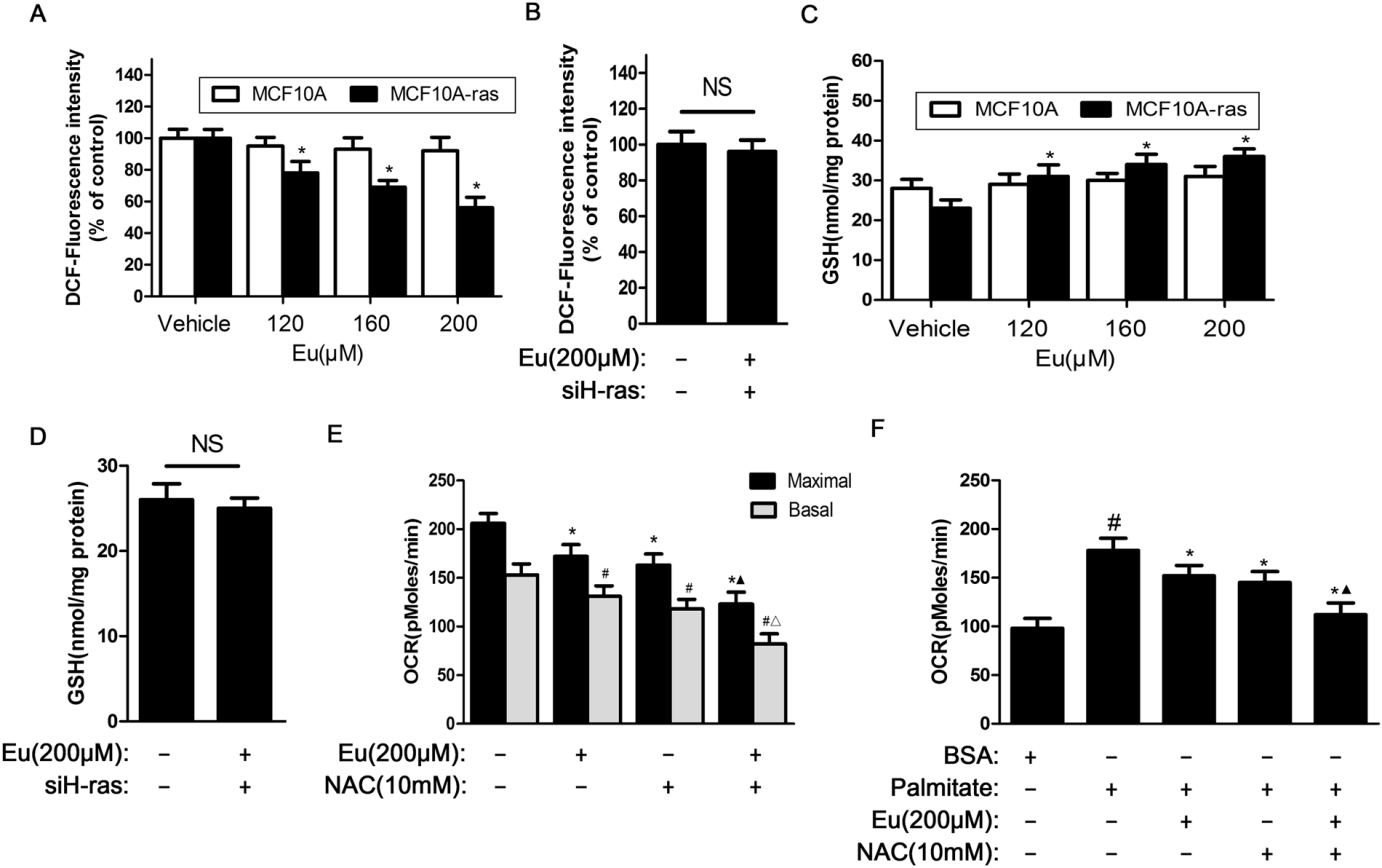
Eu inhibits OXPHOS and FAO by decreasing oxidative stress in MCF10A-ras cells. (**A**–**D**) MCF10A-ras or MCF-10A cells were treated with different concentrations of Eu for 24 h. In some experiments, MCF10A-ras cells were transfected by siH-RAS, then the cells were treated with 200 μM Eu. The fluorescence intensity and GSH content were detected by a microplate reader. Error bars represent means ± S.D. *P < 0.05 versus non-treated control of MCF10A-ras cells. NS indicates no significant difference to control. (**E**,**F**) MCF10A-ras cells were treated with 200 μM Eu, 10 mM NAC and 200 μM Eu + 10 mM NAC for 24 h. (**E**) The basal and maximal OCR were measured by Seahorse XF96 Analyzer. Data was shown as the mean ± S.D. *P < 0.05 compared with maximal OCR control group, ^#^ *P* < 0.05 compared with basal OCR control group, ^▲^ *P* < 0.05 compared with Eu-treated maximal OCR control group, ^△^ *P* < 0.05 compared with Eu-treated basal OCR control group. (**F**) OCR was measured after 200 μM palmitate injection over 2 to 3 h and cells treated with BSA were served as a negative control. Data was shown as the mean ± S.D. ^#^ *P* < 0.05 compared with vehicle control group, *P < 0.05 compared with palmitate control group, ^▲^ *P* < 0.05 compared with palmitate and Eu-treated control group.

The production of reactive oxygen species (ROS) in epithelial cancer cells is a significant liability of oxidative metabolism in that it can lead to tumor evolution, via the positive selection of tumor cell mutations that confer a growth advantage^34^. To explore whether Eu affected cellular energy metabolism which was associated with oxidative stress, we treated MCF10A-ras and MCF-10A cells with different concentrations of Eu and tested ROS and glutathione (GSH) levels. As indicated in Fig. 7A, the levels of ROS were reduced by 22.5%, 31.6% and 44.3% when MCF10A-ras cells were treated with 120, 160, 200 μM concentrations of Eu, respectively. However, Eu did not change the levels of ROS in MCF10A cells. In contrast, treatment with different concentrations (120, 160, 200 μM) of Eu led to 1.37-, 1.42- and 1.56-fold increase in GSH production in MCF10A-ras cells, and also did not affect the levels of GSH in MCF10A cells ((Fig. 7C). We further tested whether deletion of H-ras would influence ROS and GSH levels in MCF10A-ras cells treated with Eu. As a result, Eu-triggered decrease in ROS levels and Eu-induced increase in GSH levels were reversed by siRNA-mediated knock down of H-ras ((Fig. 7B,D). Next, to determine whether Eu inhibited OXPHOS and FAO through decreasing the generation of ROS, we used the N-acetyl cysteine (NAC) [10 mM], a powerful antioxidant. As shown in Fig. 7E, the maximal OCR was decreased by 16.5%, 20.8%, 40.2%, and the basal OCR was reduced by 14.3%, 22.8%, 46.5% in MCF10A-ras cells treated with 200 μM Eu, 10 mM NAC and 200 μM Eu combined with 10 mM NAC, respectively. In addition, the OCR was also respectively decreased by 14.6%, 18.5%, 37.0% in the presence of palmitate (Fig. 7F). Therefore, Eu and NAC can synergistically decrease the level of OXPHOS and FAO in MCF10A-ras cells. These findings indicate that the regulation of ROS levels by H-ras plays an important role in Eu-induced suppression of OXPHOS and FAO.

## Discussion

**Figure 8:**
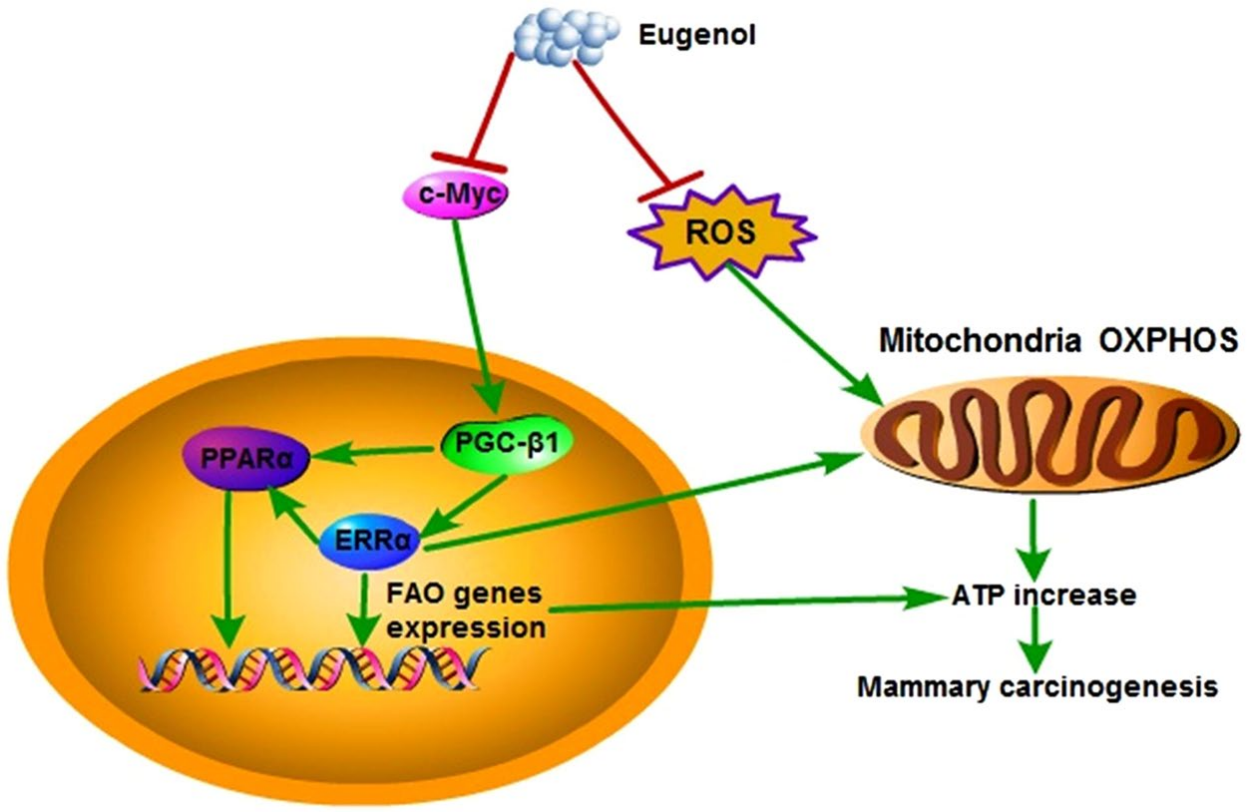
Eu reduces ATP generation and inhibits oxidative phosphorylation (OXPHOS) and fatty acid oxidation (FAO) via downregulating of c-Myc/PGC-1β/ERRα signaling pathway and inhibiting ROS production in H-ras transfected MCF10A breast epithelial cells.

Alteration in cellular energy metabolism is now firmly established as a hallmark of cancer, which promotes cell survival by increasing energy production, biomass production, maintaining redox balance, as well as through initiation of signal transduction controlled by changes in cellular metabolism^4,35,36^. Interventions targeting the metabolic pathways provide potential preventive or therapeutic methods for cancer^37,38^. In the present study, the effects of Eu on cellular energy metabolism were investigated in untransformed and H-ras transformed MCF10A cells, a human breast epithelial cell model for studying early breast cancer progression. The results support the hypothesis that Eu reduces ATP production of cells in progression to cancer by downregulating c-Myc/PGC-1β/ERRα signaling pathway and inhibiting ROS production, as a consequence of the shift in energy metabolism toward reduced OXPHOS and FAO activity in H-ras transformed MCF10A cells, suggesting a preventive effect of Eu on energy production for rapid cell proliferation during mammary carcinogenesis (Fig. 8). These results show that Eu regulates cellular energy metabolism which may be a potential mechanism for preventing early mammary carcinogenesis.

The c-Myc is a proto-oncogene which plays critical role in breast tumorigenesis and progression and has been found overexpressed in 45% of breast tumors^28^. It is involved in a variety of biological processes, including cell proliferation, differentiation, and apoptosis. It also regulates glycolysis and mitochondrial biogenesis to control cellular metabolism^17^. Ras enhances the accumulation of c-Myc activity by stabilizing the c-Myc protein during the initial stage of cell proliferation^29^. In addition, transgenic mice strains that crossed the MMTV/v-Ha-ras and MMTV/c-myc manifested a synergistic action of these two oncoproteins in accelerating mammary tumor formation^39^. It has been reported that c-Myc is amplified in the MCF10A cell line^40,41^, which demonstrates a collaborative nature of H-ras and c-Myc aberrant activity to initiate breast cancer. The present study suggests that Eu may alter energy metabolism during early breast cancer progression mediated by the H-ras oncogene. PGC-1β and ERRα are key regulators of energy metabolism, and PGC-1β is the most important coactivator for ERRα activity in breast cancer cells. It has recently been demonstrated that PGC-1β is a direct target of c-Myc, which raises the possibility that some of these well-established functions of c-Myc may be mediated by PGC-1β and ERRα^20,42^. The current study demonstrates that Eu treatment in MCF10A-ras cells but not MCF10A cells reduced ATP production in a dose-dependent manner, resulted in the decreased expression of c-Myc, PGC-1β and ERRα protein levels, suggesting Eu almost has no side-effects on normal cells. It has been suggested that most of the chemopreventive effects of Eu against different cancer cells is associated with apoptosis^23,26,43^. However, Eu induced the reduction of ATP utilization of oxidative stress in oral squamous cell carcinoma, which suggests the induction of non-apoptotic cell death by Eu^44^. Consistently, the current study showed that Eu reduced intracellular ATP levels of MCF10A-ras cells and inhibited c-Myc/PGC-1β/ERRα pathway, indicating Eu may alter energy metabolism of MCF10A-ras cells through the regulation of c-Myc/PGC-1β/ERRα pathway.

Several recent reports questioned the conventional concept that cancer cells were considered to depend only on glycolysis for ATP production, even in the presence of adequate oxygen, without major contribution from mitochondrial respiration^45^. However, it is now clear that there are multiple metabolic phenotypes, going beyond the increased glycolysis as observed by Warburg. One of these metabolic phenotypes is upregulated mitochondrial oxidative phosphorylation in human breast tumors which have been shown to have functional mitochondria capable of producing ATP^8,46^. As such, the targeting of mitochondrial OXPHOS is emerging as a novel strategy in the treatment of breast cancer^47^. Metformin and phenformin,the anti-diabetic biguanides, have been shown to exert their anticancer effect via inhibiting mitochondrial complex I, the first enzyme in the mitochondrial electron respiratory chain^48,49^. Previous studies reveal that the H-ras oncoprotein alone may lead to an increase in mitochondrial metabolism in human cells, similar to that seen in cancer cells^50^. The MCF10A-ras cells, an initiation stage of breast cancer progression, show changes in several metabolism status, including increased carbon flux through the pentose phosphate pathway (PPP), the TCA cycle, as well as increased synthesis of fatty acids, supporting the hypothesis that very early changes in metabolism status may occur during cancer progression in the presence of the H-ras oncogene^7,50^^.^ In the MCF10A-ras cells, Eu was shown to severely reduced intracellular ATP levels and OCR. Surprisingly, the proteins expression of NDUFB8, SDH-B, COX5A, UQCRC2, ATP5A, which represent mitochondrial complex I-V, were decreased by Eu in the MCF10A-ras cells. One mechanism that may contribute to the reduction of these complex proteins may be due to the reduced activity of the upstream PGC-1β/ERRα axis in respond to Eu. It has been suggested that PGC-1 plays a critical role in the control of multiple aspects of energy metabolism, including mitochondrial biogenesis, fatty acid β-oxidation and adaptive thermogenesis^51^. This family is composed of three members, PGC-1α and PGC-1β, and PGC-related coactivator (PRC). However, PGC-1β, but not PGC-1α or PRC, was most closely associated with ERRα in breast cancer cell^20^. In our study, the overexpression of PGC-1β nearly blocked any Eu-induced downregulation of mitochondrial complex proteins levels. ERRα is a member of the nuclear hormone receptor superfamily of transcription factors and plays a role in energy homeostasis and will probably be a novel target for the future development of breast cancer. Expectedly, through inhibition ERRα-modulated activity of electron transport chain complex, Eu reduced ATP production and OCR in the MCF10A-ras cells, which was confirmed by siRNA-mediated silencing of ERRα. from these data, we conclude that Eu induce inhibitory effects of OXPHOS through PGC-1β/ERRα axis.

In the past decade, the large majority of the research into cancer metabolism has been limited to a handful of metabolic pathways, while other pathways still remain unclear^52^. Recently, alternative energy pathways like FAO have been attracted increasing attention in cancer research^53,54^. To verify the role of Eu in lipid catabolism, expression of the genes involved in FAO were examined. Indeed, the expression of genes related to FAO, such as PPARα, CPT1C, and MCAD, were regulated by transcriptional factor ERRα^42,55,56^. It was also observed that the FAO levels and protein expression of PPARα, MCAD and CPT1C were obviously decreased in MCF10A-ras cells after the treatment with Eu. Highly consistent with our preceding results, PGC-1β/ERRα axis is required for inhibition of FAO levels and its proteins by Eu. It has been well-established that regulation of FAO mainly involves CPT1 in normal cells^57^. CPT1C might be supplementing the high energy needs of cancer cells via FAO, and that cancers lacking CPT1C could not take advantage of this supplementation, and thus were more sensitive to metabolic stress^10^. Consistent with this hypothesis, breast cancer cell line constitutively expressing CPT1C show increased FAO, ATP production, and resistance to glucose deprivation or hypoxia^33^. Additionally, mitochondrial free fatty acid β-oxidation is certainly functional in cancer cells, driving OXPHOS-dependent ATP supply for the accelerated cell proliferation^58^. Our results indicate that inhibition of FAO by Eu may contribute to the growth inhibitory effects in MCF10A-ras cells.

Oxidative stress plays an important role in the initiation and progression of breast cancer. H-ras oncogene activation in epithelial cancer cells drives oxidative stress and induces ROS production. H-ras oncogene-induced ROS production can also promote increases in mitochondrial activity in epithelial cancer cells. Thus, H-ras oncogene drives the metabolic reprogramming of cancer cells via oxidative stress. Moreover, antioxidants can selectively halt mitochondrial biogenesis in H-ras transformed epithelial cancer cells^59,60^. In the present study, treatment with Eu inhibited OXPHOS and FAO through decreasing ROS levels in MCF10A-ras cells. Our work have shown that Eu has protective effects on MCF-10A cells. Likewise, Eu has less influence on the cellular ROS of MCF-10A cells than MCF10A-ras cells. On the basis of all above, our results suggest that the OXPHOS and FAO are related to the high level of cellular ROS in MCF10A-ras cells, which is a potential target for prevention of breast cancer.

The Eu has been proven to be a safe and effective chemopreventive agent through multiple functions during early breast cancer progression, which preferentially kills the malignant cells that are dependent on OXPHOS and FAO pathway for ATP generation. Results from our laboratory show that Eu may effectively alleviate or inhibit the progress of breast precancerous lesions and the occurrence rate of breast precancerous lesions in the rat models induced by dimethylbenzanthracene (DMBA) combined estrogen and progesterone (data not shown). Our work provides a novel promising therapeutic regimen for clinical practice and a strong theoretical basis for subsequent clinical trials. To deliver Eu to breast tumor *in vivo* or in clinical practice for high efficacy but low toxicity, two aspects need to be considered. On the one hand, the susceptibility of patient’s tumor to Eu therapy needed to be determined by evaluating glucose utilization level and the expression of glucose transporters of the tumor before treatment^61,62^. On the other hand, an assay or test to detect energy state (ATP levels) in the tumor should be performed to adjust dosage regimens.

In conclusion, these results of the current study suggest that Eu reduces ATP generation and inhibits OXPHOS and FAO by downregulating c-Myc/PGC-1β/ERRα pathways and inhibiting ROS production in H-ras transfected MCF10A breast epithelial cells. To our knowledge, this is the first study to demonstrate that Eu inhibits cell proliferation and/or survival through regulating cellular energy metabolism during early mammary carcinogenesis. Therefore, administration of natural compound eugenol to inhibit, block, reverse or delay the initiation and promotional events associated with mammary carcinogenesis opens a new avenue for cancer prevention to reduce breast cancer morbidity.

## Materials and Methods

### Reagents

The Eu was purchased from National Institutes for Food and Drug Control (China). Dulbecco’s modified Eagle medium (DMEM), Nutrient Mixture F-12 (DMEM/F12) media, horse serum, trypsin and penicillin/streptomycin, were obtained from Life Technologies, GibcoBRL (Rockville,MD,USA). Antibodies against CPT-1C and Tomm20 were purchased from Santa Cruz Biotechnology (Santa Cruz, CA,USA). Antibodies against ERRα and c-Myc were obtained from Cell Signaling Technology (Beverly, MA,USA) and an antibody against the HRAS, PGC-1β, NDUFB8, SDH-B, UQCRC2, ATP5A, COX5A, MCAD and PPARα were obtained from Abcam (Cambridge, MA, USA). 10058F4 were purchased from Sigma-Aldrich (St.Lois, MO, USA). XF Palmitate-BSA FAO Substrate was obtained from seahorse Bioscience (North Billerica, MA, USA).

### Cell lines

MCF10A, MCF-7, BT-474 and SKBR-3 cell lines were purchased from the American Type Culture Collection (ATCC, Manassas, VA, USA) and cultured according to manufacturer’s directions. MCF10A-ras cell lines were obtained from American Karmanos cancer institute. MCF10A and MCF10A-ras cells were cultured in DMEM/F12 (1:1) supplemented with 5% horse serum, 10 μg/ml insulin, 50 μg/ml hydrocortisone, 20 ng/ml recombinant epidermal growth factor, 100 units/ml penicillin, and 0.1 mg/mL streptomycin, at 37 °C, in a humidified 5% CO2 atmosphere. Cells were maintained in linear growth. Eu was delivered to cells in 100% dimethyl sulphoxide (DMSO) at a final DMSO concentration <0.5%, and it had no effect on MCF-10A and MCF10A-ras cells.

### Cell viability assay

Cell viability was measured using the MTT (3-(4,5-dimethylthiazol-2-yl) -2,5-diphenyl tetrazolium bromide) assay. Briefly, cells were seeded at a density of 5000 cells/well in a 96-well plate and incubated overnight. The medium was replaced with fresh one containing the desired concentration of Eu. After 24 h, 20 μL of MTT solution (5 mg/ml stock solution, Sigma) was added to each well and the plates were incubated for 2–4 h at 37 °C. The medium was removed and the formazan blue, which formed in the cells, was dissolved in 100 μL of DMSO. The absorbance was read at 570 nm using a microplate reader. Cell viability was calculated as percentage of control (untreated or vehicle-treated cells) and averaged from three independent experiments.

### Measurement of intracellular ATP

Intracellular ATP was measured using a firefly luciferase-based ATP assay kit (Beyotime, Jiangsu, China) according to the manufacturer’s instructions. Briefly, cells were cultured in DMEM with indicated treatments. After 24 h, cells were lysed and centrifuged at 12,000g for 5 min. The supernatants (100 μl) were mixed with 100 µL of ATP detection working dilution in a white 96-well plate. Luminescence was measured by a microplate reader (Bio-Tek Instruments, Inc, Winooski, VT, USA). Standard curves were also generated according to the protocol of the ATP assay kit. This experiment was repeated for three times.

### Western blot analysis

Cells were washed with ice-cold PBS, harvested and extracted with lysis buffer at 4 °C. Protein concentration was determined using the BCA assay (Beyotime, Jiangsu, China). The protein lysates separated by 12% SDS-PAGE gel were electrophoretically transferred to polyvinylidene difluoride (PVDF) membranes (Millipore, Billerica, MA, USA) and subsequently incubated in blocking buffer (5% nonfat dry milk) for 2 h at room temperature. The membranes was incubated with primary antibody (diluted according to the manufacturer’s instructions) overnight at 4 °C. The next day, the membranes were incubated with 1:5000 dilution of horseradish peroxidase-conjugated secondary antibody for 45 mins at room temperature. After extensive washes with 0.05% Tween-20/PBS, the labeled protein spots were detected by chemiluminescence detection.

### Metabolic assays

Oxygen consumption rate (OCR) was determined using the Seahorse XF96 extracellular flux analyzer (Seahorse Bioscience, North Billerica, MA, USA) as previously described^63^. Briefly, 2 × 10^4^ MCF10A-ras cells per well were seeded overnight in XF96 well culture microplates in a 37 °C /5% CO2 incubator. One hour prior to assay, cells were equilibrated with unbuffered DMEM and incubated at 37 °C for pH and temperature stabilization in a non-CO2 incubator. Analyses were performed both at basal conditions and after injection of Oligomycin (1 μM), FCCP (0.6 μM), Antimycin A/rotenone (1 μM each) at indicated time points. All data were analyzed using XF software and displayed as average OCR (pM/min).

### Fatty acid oxidation assay

Similarly to above, 4 × 10^4^ cells were seeded in XF96 well culture microplates. At 24 h prior to analysis, culture media was replaced by substrate limited DMEM media (0.5 mM glucose, 1 mM Glutamine, 0.5 mM carnitine, and 1% FBS) and incubated at 37 °C to determine the ability of cells to oxidize exogenous fatty acids. On the day of assay, the FAO assay KHB buffer (110 mM NaCl, 4.7 mM KCl, 2 mM MgSO_4_, 1.2 mM Na_2_HPO_4_, 2.5 mM glucose adjusted to pH 7.4) supplemented with 0.5 mM carnitine and 5 mM HEPES was exchanged in 37 °C incubator. For induction of FAO, BSA conjugated palmitate was injected to a final concentration of 50 μM. Basal OCR of cells treated with palmitate coupled to BSA or BSA vehicle alone were measured.

### Transfection of PGC-1β plasmids

Cells were seeded in 6-well plates at 65% confluency at first. Then, pcDNA3.1-PGC-1β was introduced into the cells using Lipofectamine 2000 (Invitrogen Life Technologies, Carlsbad, CA, USA) according to the manufacturer’s protocol. At 24 h post transfection, cells were treated with Eu or the vehicle and cultured for an additional 24 h, prior to collection for western blot analyses.

### siRNA Experiments

Cells were transfected using Lipofectamine 2000 according to the manufacturer’s protocol with 50 nM HRAS or ESRRA siRNA. After formation of the liposome–siRNA mixture, the complexes was added to various breast cancer cells and MCF10A-ras cells for 4 h. The medium was then replaced with fresh medium containing 200 μM Eu.

### Measurement of intracellular ROS

The intracellular ROS level was measured by using 2,7-dichlorofluorescin diacetate (DCFH-DA) (Beyotime, Jiangsu, China). The DCFH-DA is an oxidation-sensitive fluorescent probe. Briefly, 10 mM DCFH-DA stock solution was diluted in culture medium without serum to yield a 10 μM working solution. After treatment with different concentrations of eugenol for 24 h, cells were washed twice with PBS. Then they were incubated in 10 μM working solution of DCFH-DA at 37 °C for 30 min. The intensities of fluorescence were measured at excitation 488 nm and emission 525 nm using a multi-mode microplate reader (Bio-Tek Instruments, Inc, Winooski, VT, USA). The values were expressed as percent of fluorescence intensity relative to control wells.

### Intracellular GSH Content Assay

Glutathione (GSH), an antioxidant, protects cells from free radicals. Intracellular reduced GSH levels were quantified by the Reduced Glutathione Assay Kit (Nanjing Jiancheng Bioengneering Institute, Nanjing, China). Briefly, cells were treated with different concentrations of eugenol for 24 h. Following treatment, cells were then harvested and washed with PBS. The intracellular GSH was assayed by measuring the absorbance of cell extract at 412 nm using a multi-mode microplate reader (Bio-Tek Instruments, Inc, Winooski, VT, USA) according to the kit instructions. GSH levels were estimated after normalization to cellular protein levels and expressed as GSH nmol/mg protein.

### Statistical analysis

All the values presented are expressed as mean ± SD. Comparison of different groups was carried out using the two-tailed unpaired student *t* test. *P* values less than 0.05 (*P* < 0.05) were considered statistically significant.

## Electronic supplementary material

Supplementary Information
