# Supplementary material for: Eugenol inhibits oxidative phosphorylation and fatty acid oxidation via downregulation of c-Myc/PGC-1β/ERRα signaling pathway in MCF10A-ras cells
Source: Sci Rep. 2017 Oct 10;7:12920. doi: 10.1038/s41598-017-13505-x (PMC5634997; doi:10.1038/s41598-017-13505-x)
Supplement: Supplementary file 1 — Supplementary Information [file 41598_2017_13505_MOESM1_ESM.pdf]

Supplementary Information

Eugenol inhibits oxidative phosphorylation and fatty acid oxidation via downregulation of c-Myc/PGC-1β/ERRα signaling pathway in MCF10A-ras cells

Xianxin Yan[1,5], Guijuan Zhang[2,5], Fengjie Bie[1,5], Yanhong Lv[1], Yi Ma[3], Min Ma[1,4], Yurong Wang[1], Xiaoqian Hao[1], Naijun Yuan[1], Xuefeng Jiang[1]

[1]College of Traditional Chinese Medicine, Jinan University, Guangzhou, China. [2]The School Outpatient Department, the First Affiliated Hospital of Jinan University, Guangzhou, China. [3]Bio-engineering institute of Jinan University, Guangzhou, China.

[4]Correspondence and requests for materials should be addressed to M.M.

-The Full Postal address: College of Traditional Chinese Medicine of Jinan University, Institute of Integrated Traditional Chinese and Western Medicine of Jinan University, 601 Huangpu Ave. West, Guangzhou 510632, Guangdong Province, China. Guangzhou, CN 510632

-The Email Address: tmamin@jnu.edu.cn.

-Telephone: 00862085227137

-FAX: 00862085221983

[5]Co-first authorship.

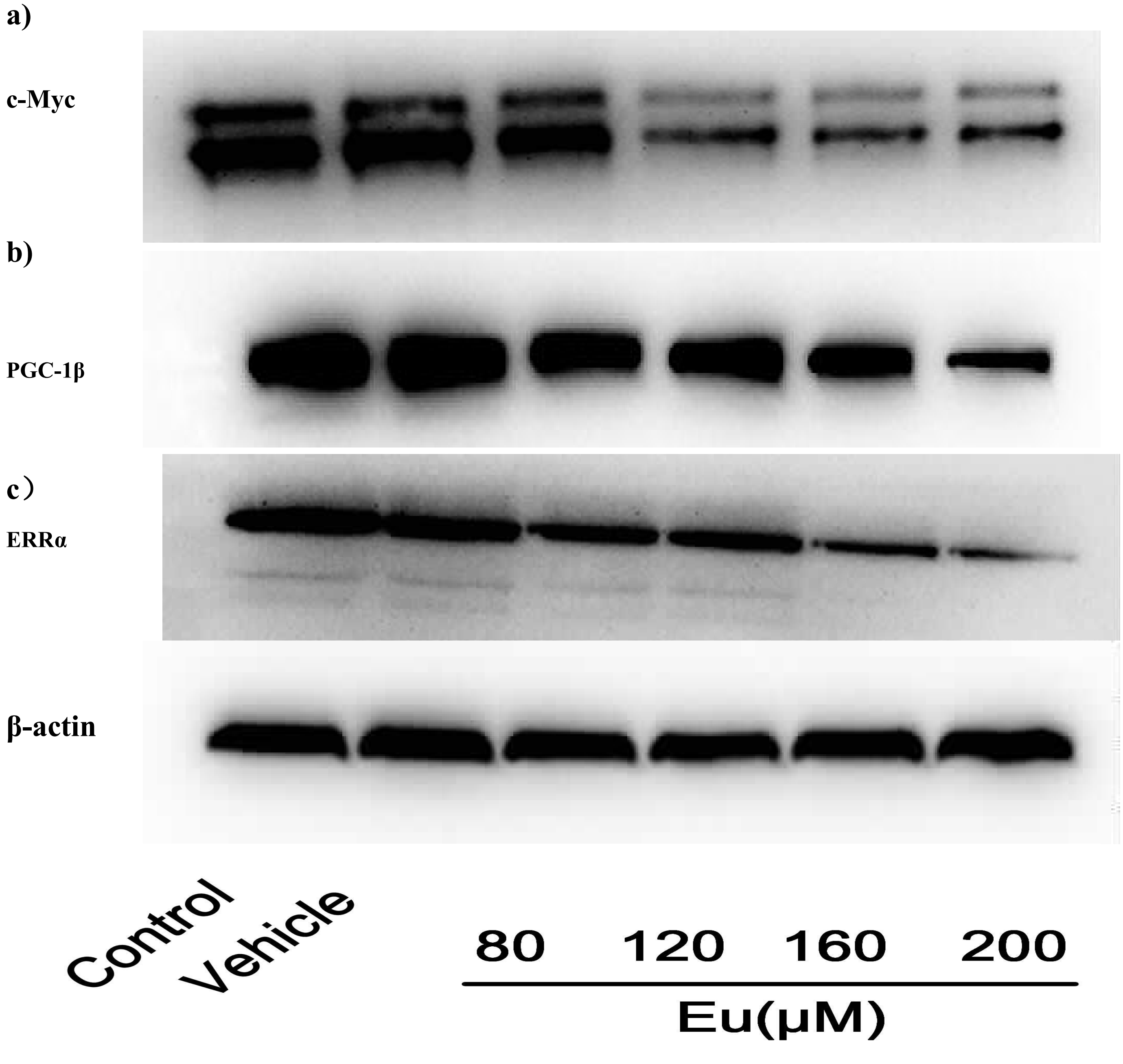

Supplementary Figures S1：Western blots showing the different protein expression levels of c-Myc, PGC-1β and ERRα in MCF10A-ras cells treated with different concentrations of eugenol.

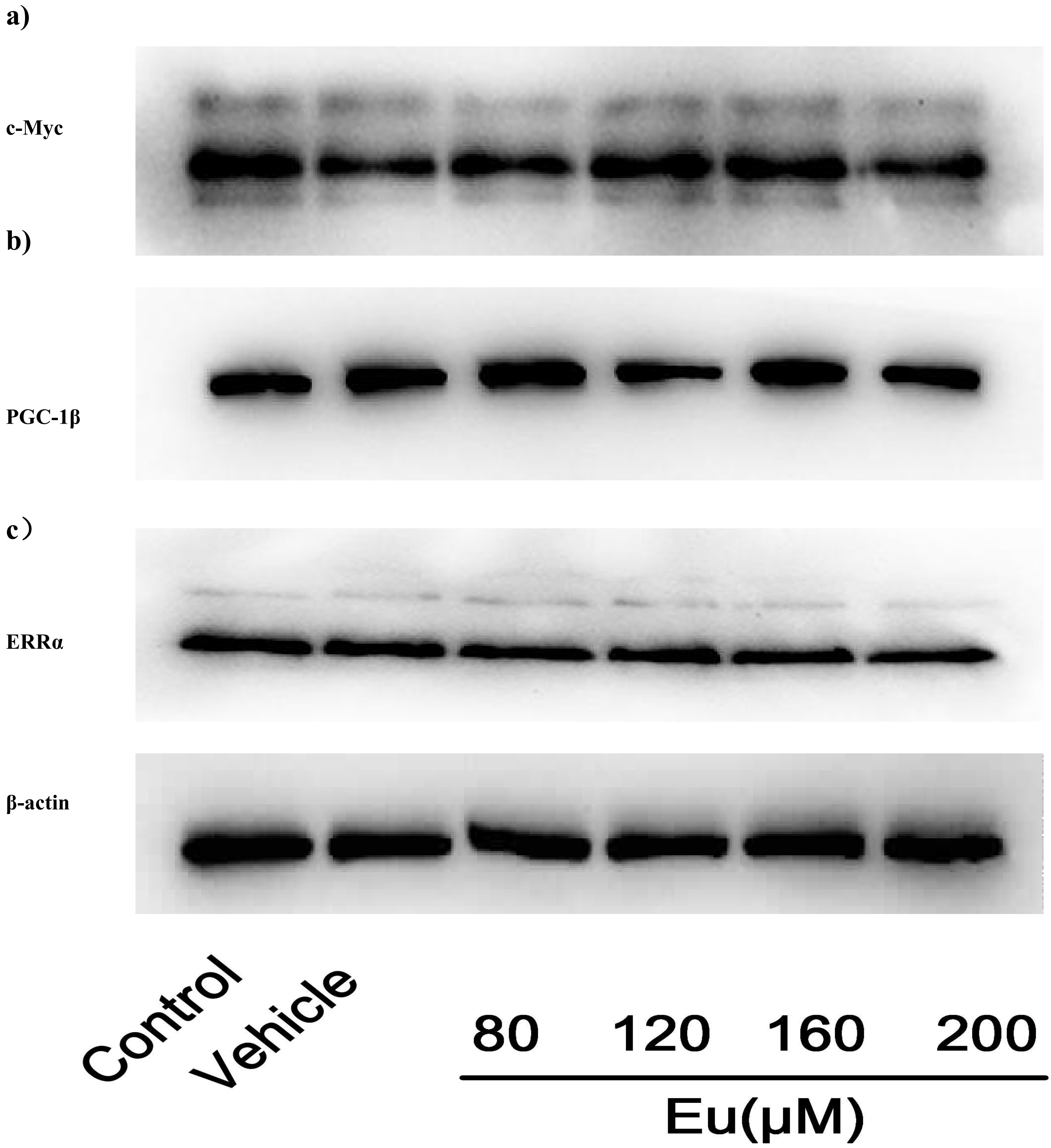

Supplementary Figures S2：Western blots showing the different protein expression levels of c-Myc, PGC-1β and ERRα in MCF10A cells treated with different concentrations of eugenol.

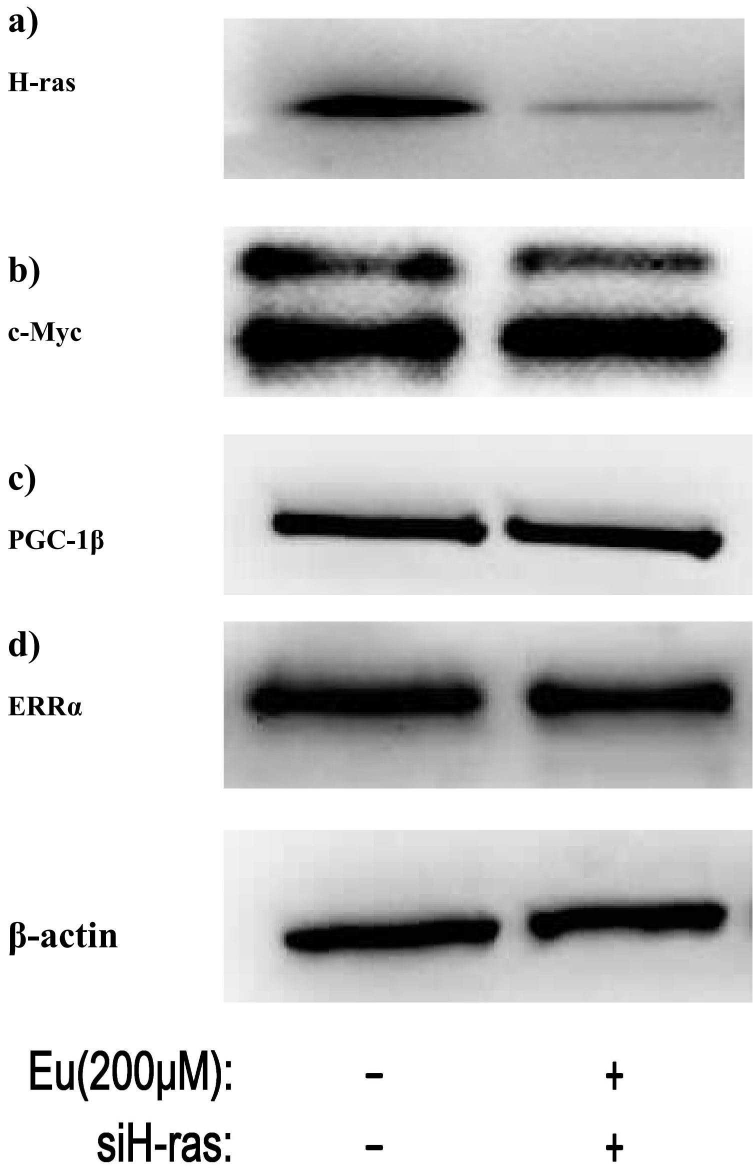

Supplementary Figures S3：MCF10A-ras cells were transfected with siH-RAS, then the cells were treated with 200 μM Eu. The protein expression levels of H-ras, c-Myc, PGC-1β and ERRα were detected by western blot.

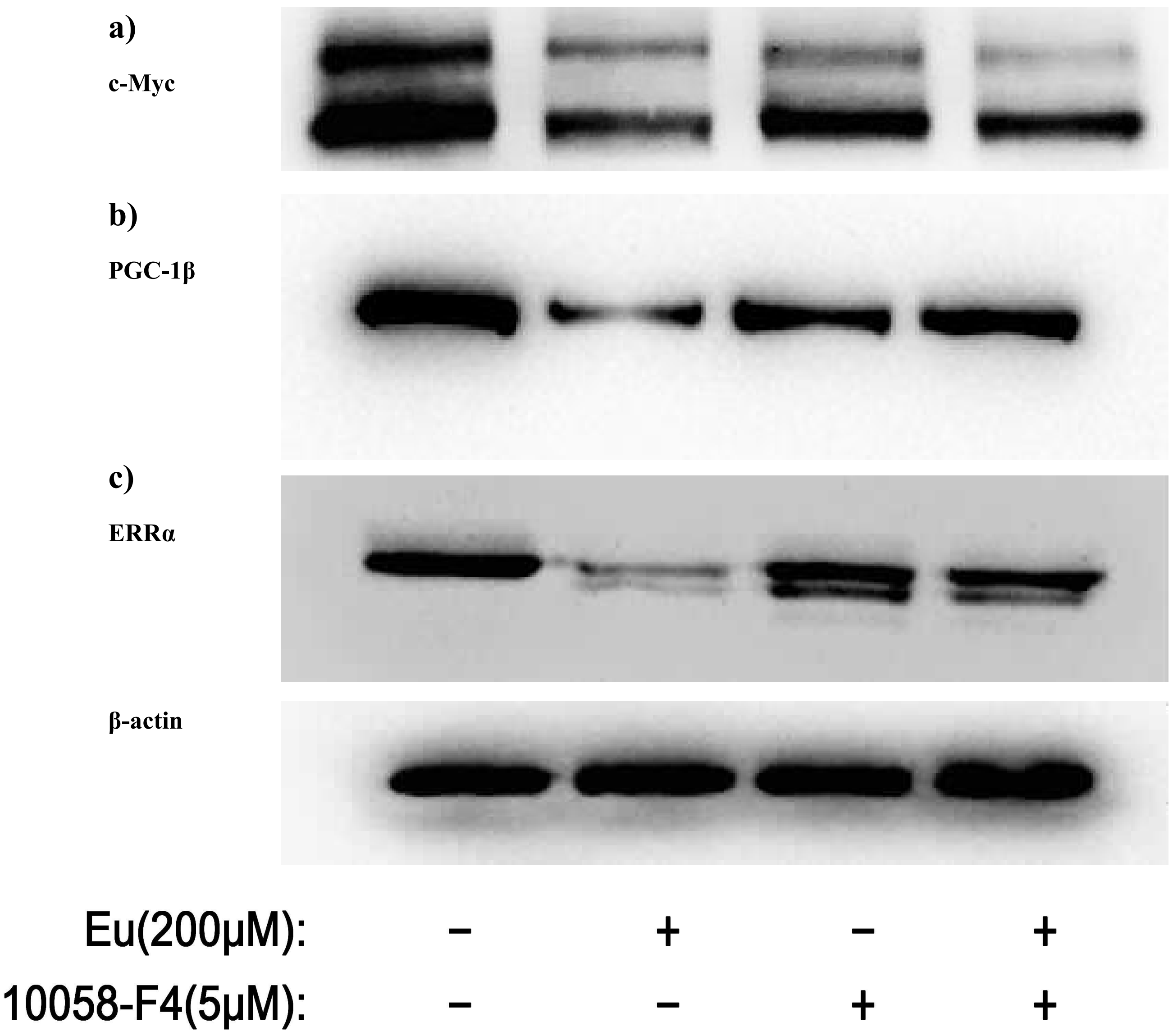

Supplementary Figures S4: Western blots showing the protein expression levels of c-Myc, PGC-1β and ERRα in MCF10A-ras cells in different treatment groups (Vehicle, Eu, 10058-F4 and 10058-F4+Eu).

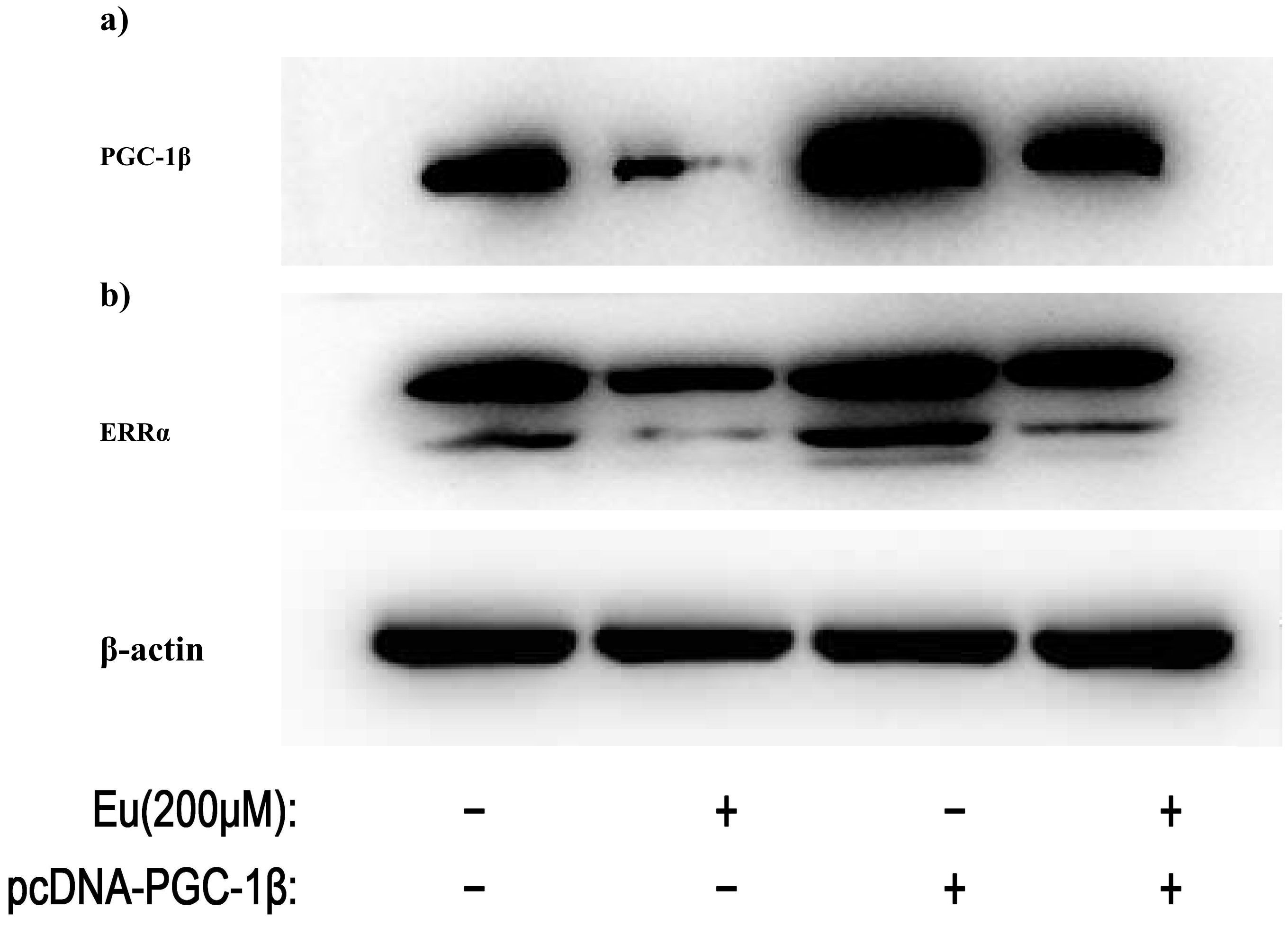

Supplementary Figures S5: Western blots showing the protein expression levels of PGC-1β and ERRα in MCF10A-ras cells in different treatment groups (Vehicle, Eu, pcDNA-PGC-1β and pcDNA-PGC-1β+Eu).

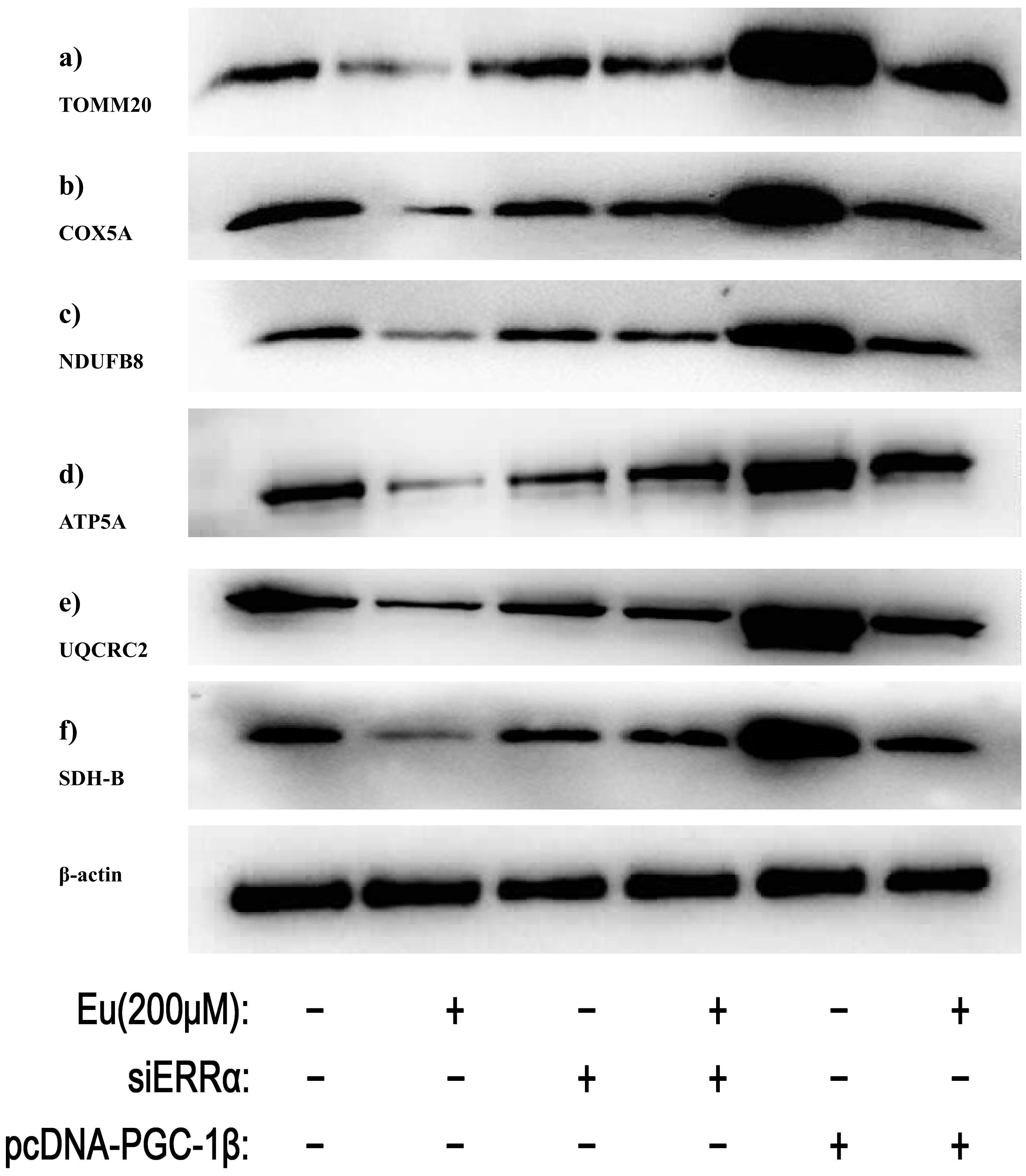

Supplementary Figures S6: Western blots showing the protein expression levels of mitochondrial complexes (COX5A, NDUFB8, ATP5A, UQCRC2, SDH-B) and TOMM20 in MCF10A-ras cells in different treatment groups (Vehicle, Eu, siERRα, siERRα+Eu, pcDNA-PGC-1β, pcDNA-PGC-1β+Eu).

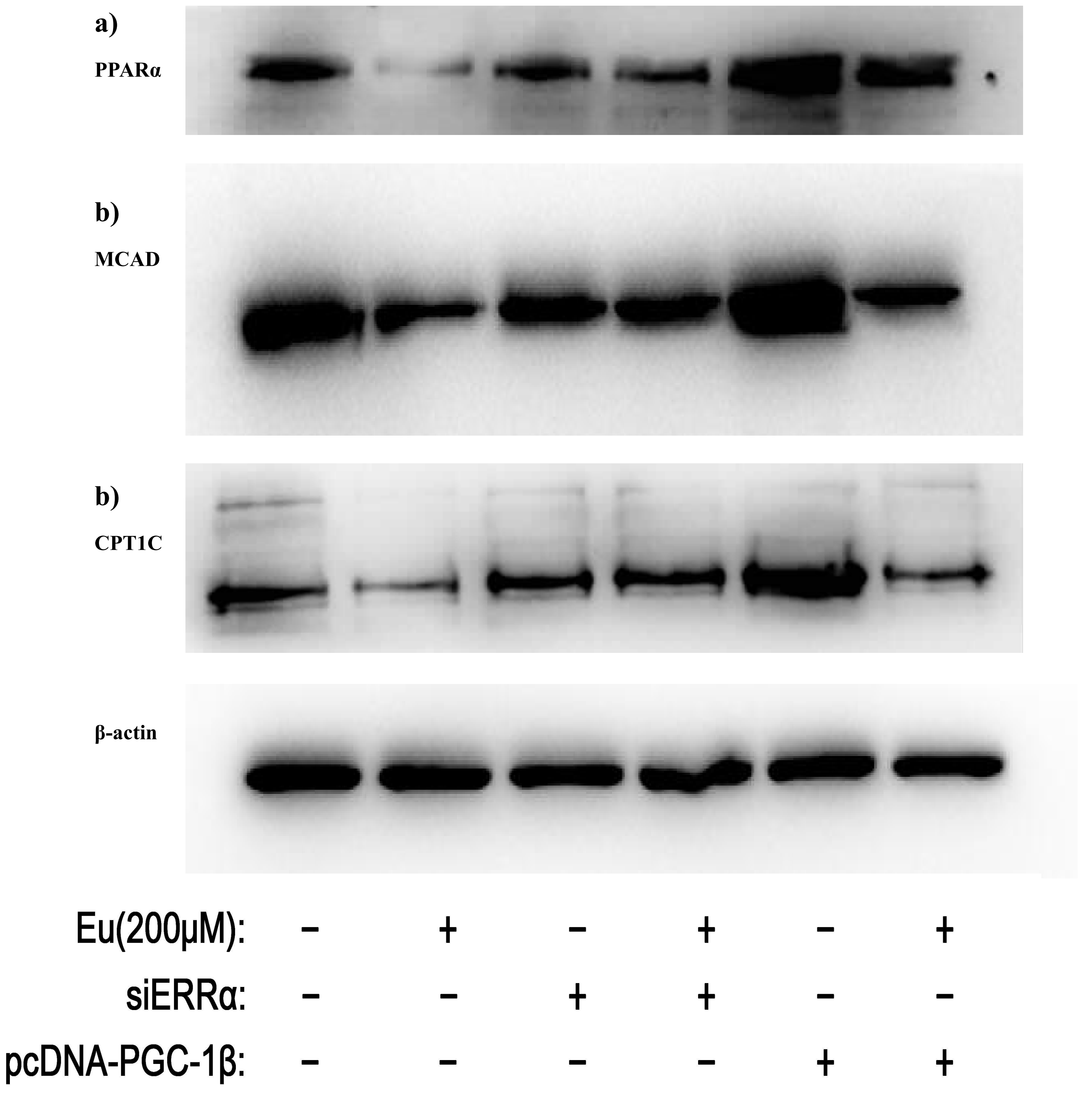

Supplementary Figures S7: Western blots showing the protein expression levels of PPARα, MCAD and CPT1C in MCF10A-ras cells in different treatment groups (Vehicle, Eu, siERRα, siERRα+Eu, pcDNA-PGC-1β, pcDNA-PGC-1β+Eu).
